# St. Louis Journal of Homœopathy

**Published:** 1895-01

**Authors:** 


St. Louis Journal of Homœopathy. W. A. Edmonds, A. M., M. D., Editor, No. 2924 Washington Ave., St. Louis, and J. Martine Kershaw, M. D., Managing Editor, St. Louis, Mo. The Shultz Publishing Co., 105 and 107 North Sixth Street. Subscription price, $1.00 a year.

The first number of this new monthly magazine, issued for the month of December, is now before the profession. It is a pamphlet of thirty-two pages full of interesting medical articles and some portraits of the professors in the Homœopathic Medical College of Missouri.

In his "Salaam" the editor says: "The study and practice of medicine the world over, in all the schools, is practically a unit except in the matter of *therapeutics.* The time is past when any body of scientific men may sit in judgment and proclaim itself the repository of all truth, and that its neighbors and others, equally sincere and able, are but fools or frauds. This admission and declaration may not be taken that I propose to yield one jot or tittle in behalf of Hahnemann's wise and wonderful rule governing the selection of the suitable single remedy in its minimum dose in the treatment of disease."

